# Supplementary material for: Spatial spillover and impacting factors of green development: A study based on China’s provincial data
Source: PLoS One. 2023 Mar 15;18(3):e0282865. doi: 10.1371/journal.pone.0282865 (PMC10016718; doi:10.1371/journal.pone.0282865)
Supplement: Attachment — Submitted filename: Response Letter-plos one.docx [file pone.0282865.s002.docx]

**Response to Reviewer 1**

**Comment #1:**

This paper studies spatial spillover and impacting factors of green development based on China’s provincial data. The subject is interesting, but the overall quality of the paper shall be improved before publication.

**Response #1:**

We wish to thank R1 for the encouraging comment. We have carefully revised the manuscript according to R1’s suggestions.

**Comment #2:**

Major points:

In the past two years, there have been many literatures on green development and space spillovers. It is suggested to add in the literature review.

**Response #2:**

Thanks for this suggestion. We have added a few recent publications on green development and space spillovers in the revised manuscript. Specifically, we organize the relative literature into three aspects: 1) research on development heterogeneity of green development; 2) impact factors of green development; 3) spatial interdependence and spillover of green development. See the content below:

*“Recent research on regional green development can be divided into several aspects around development heterogeneity. First, researchers have focused on establishing an evaluation system of green development levels and efficiencies. Some researchers have developed operational measurements of regional green development level based on the analytic hierarchy process (AHP) entropy method [2-3], the three-dimensional AHP method [4], the entropy technique for order preference by similarity to ideal solution model in combination with the Theil index method [19,20], the multi-hierarchy linear summation method [21], or using a software tool to assess energies in multi-scale life cycles [22]. Other researchers have focused on establishing the green total factor productivity to depict the green development efficiency among different regions. For instance, researchers have applied data envelopment analysis, including the non-radial direction distance function method [5], the slacks-based measure (SBM) model with undesirable outputs [7], and the SBM Malmquist–Luenberger model to evaluate the green total factor productivity [7,23,24]. By evaluating those green development indexes, researchers have found an imbalance in green development among different regions. Specifically, eastern China has outpaced central and western China in green development [4,8-10], and certain cities have higher green development levels or efficiencies than other cities within the same region [6,7,19-21, 25].*

*Researchers have identified a range of impact factors of green development, with research on the effect of green development policy, especially environmental regulations, being particularly notable. The Chinese government has put forward environmental regulations in relation to both command-control and market-related policies, prioritizing sustainable development strategies. Different environmental regulations may affect green development unequally [11,13,26]. Variations in policy effectiveness may be one reason the literature offers diverse viewpoints regarding the effects of environmental regulations. For instance, Pan et al. [27] stated that environmental regulations contribute to technological advancement and energy conservation. Jiang et al. [28] found that environmental regulations function negatively for green development, inhibiting enterprises’ R&D investment, and thus impeding their green innovation practice. Comparing US versus Swiss hydropower generation regulations, Tonka [29] proposed that defects in the regulatory framework might be the reason for environmental over-exploitation. Mycoo et al. [30] concluded that regulations are ineffective when proper regulatory tools are unavailable: a governance framework should be combined with the available effective market instruments, new technologies, behavioral change, and land tenure reform. Other factors have also been highlighted, including climatic changes and human activities [14], financial activities such as green credit and renewable energy investment [15,24,31-33], public participation and regulatory compliance [16], technical progress, and innovation [7,17,18,34,35].*

*Since the “economic geography” theory was put forward [36], the spatial correlations of economic activities of agglomeration have drawn attention in many contexts [37,38], including economic development [39,40] and financial sectors [41,42]. Spatial correlations among regions also exist in the context of green development. For instance, green environment efficiency differs across regions and shows solid spatial dependence [43,44]. This spatial interdependence is prominent in specific indicators such as carbon dioxide emissions, carbon intensity, and water resources [45-47] and in single factors like environmental regulation, technological innovation, and pollution control programs [48-50], as well as the more comprehensive green development indicators of green competitiveness [51] and green total factor productivity [52].”*

Please also refer to Line 63–109, Page 4–6 in the revised manuscript.

***References:***

2.Yang Y, Guo H, Chen L, Liu X, Gu M, Ke X. Regional analysis of the green development level differences in Chinese mineral resource-based cities. Resources Policy. 2019; 61: 261–272.

3.Guo ST, Zhang WH, Liu ZJ. Research on the temporal and spatial differentiation of the provincial green development index along the “the belt and road”. Journal of Chongqing University of Arts and Sciences. 2022; in press. https://kns.cnki.net/kcms/detail/50.1182.C.20220117.1342.002.html.

4.Li S, Liu J, Hu X. A three-dimensional evaluation model for green development: evidence from Chinese provinces along the belt and road. Environment, Development and Sustainability, 2022; in press. https://doi.org/10.1007/s10668-022-02542-w.

5.Wang W, Xie HL, Lu FC, Zhang XM. Measuring the performance of industrial green development using a non-radial directional distance function approach: A Case Study of Jiangxi Province in China. Sustainability. 2017; 9(10): e1757.

6.Wu H, Li Y, Hao Y, Ren S, Zhang P. Environmental decentralization, local government competition, and regional green development: evidence from China. Science of The Total Environment, 2020; 708: e135085.

7.Huang H, Mo R, Chen X. New patterns in China’s regional green development: An interval Malmquist–Luenberger productivity analysis. Structural Change and Economic Dynamics. 2021; 58: 161–173.

8.Lin B, Benjamin NI. Green development determinants in China: A non-radial quantile outlook. Journal of Cleaner Production. 2017; 162: 764–775.

9.Zhou L, Zhou C, Che L, Wang B. Spatio-temporal evolution and influencing factors of urban green development efficiency in China. Journal of Geographical Sciences. 2020; 30(5): 724–742.

10.Yang Y, Guo H, Chen L, Liu X, Gu M, Ke X. Regional analysis of the green development level differences in Chinese mineral resource-based cities. Resources Policy. 2019; 61: 261–272.

11.Li R, Ramanathan R. Exploring the relationships between different types of environmental regulations and environmental performance: Evidence from China. Journal of Cleaner Production. 2018; 196: 1329–1340.

12.Huang J, Zhang, W; Ruan, W. Spatial spillover and impact factors of the internet finance development in China. Physica A: Statistical Mechanics and its Applications. 2019; 527: e121390.

13.Yang J, Guo H, Liu B, Shi R, Zhang B, Ye W. Environmental regulation and the Pollution Haven Hypothesis: Do environmental regulation measures matter?. Journal of Cleaner Production. 2018; 202: 993–1000.

14.Lu X. Vulnerability of water discharge of large Chinese rivers to environmental changes: an overview. Regional Environmental Change. 2004; 4: 182–191.

15.He L, Zhang L, Zhong Z, Wang D, Wang F. Green credit, renewable energy investment and green economy development: Empirical analysis based on 150 listed companies of China. Journal of Cleaner Production. 2019; 208: 363–372.

16.Fu J, Geng Y. Public participation, regulatory compliance and green development in China based on provincial panel data. Journal of Cleaner Production. 2019; 230: 1344–1353.

17.Tao F, Zhang H, Hu J, Xia X. Dynamics of green productivity growth for major Chinese urban agglomerations. Applied Energy. 2017; 196: 170–179.

18.Li W, Wang J, Chen R, Xi Y, Liu S, Wu F, Masoud M, Wu X. Innovation-driven industrial green development: The moderating role of regional factors. Journal of Cleaner Production. 2019; 222: 344–354.

19.Wang M, Feng C. Revealing the pattern and evolution of global green development between different income groups: A global meta-frontier by-production technology approach. Environmental Impact Assessment Review. 2021; 89: e106600.

20.Wang Y, Hu H, Dai W, Burns K. Evaluation of industrial green development and industrial green competitiveness: Evidence from Chinese urban agglomerations. Ecological Indicators. 2021; 124: e107371.

21.Chen C, Ge C. Green development assessment for countries along the belt and road. Journal of environmental management. 2020; 263: e110344.

22.Allende AL, Stephan A. Life cycle embodied, operational and mobility-related energy and greenhouse gas emissions analysis of a green development in Melbourne, Australia. Applied Energy. 2022; 305: e117886.

23.Streimikis J, Miao Z, Balezentis T. Creation of climate-smart and energy-efficient agriculture in the European Union: pathways based on the frontier analysis. Business Strategy and the Environment. 2021; 30(1): 576–589.

24.Yu C, Wenxin L, Khan SU, Yu C, Jun Z, Yue D, Zhao M. Regional differential decomposition and convergence of rural green development efficiency: evidence from China. Environmental Science and Pollution Research. 2020; 27(18): 22364–22379.

25.Wang H, He D, Yu J. Privacy-preserving incentive and rewarding scheme for crowd computing in social media. Information Sciences. 2019; 470: 15–27.

26.Shen N, Liao H, Deng R, Wang Q. Different types of environmental regulations and the heterogeneous influence on the environmental total factor productivity: Empirical analysis of China’s industry. Journal of Cleaner Production. 2019; 211: 171–184.

27.Pan X, Ai B, Li C, Pan X, Yan Y. Dynamic relationship among environmental regulation, technological innovation and energy efficiency based on large scale provincial panel data in China. Technological Forecasting and Social Change. 2017; 144: 428–435.

28.Jiang Z, Wang Z, Li Z. The effect of mandatory environmental regulation on innovation performance: Evidence from China. Journal of Cleaner Production. 2018; 203: 482–491.

29.Tonka L. Hydropower license renewal and environmental protection policies: a comparison between Switzerland and the USA. Regional Environmental Change. 2015; 15: 539–548.

30.Mycoo MA, Griffith-Charles C, Lalloo S. Land management and environmental change in small-island-developing states: the case of St. Lucia. Regional Environmental Change. 2017; 17: 1065–1076.

31.Feng C, Wang M. Journey for green development transformation of China’s metal industry: A spatial econometric analysis. Journal of Cleaner Production, 2019; 225: 1105–1117.

32.Dong XJ, Shi T. The green development efficiency and influencing factors of central plains urban agglomeration. Regional Economic Review. 2018; 5: 116–122.

33.Yuan H, Zhang T, Feng Y, Liu Y, Ye X. Does financial agglomeration promote the green development in China? A spatial spillover perspective. Journal of Cleaner Production. 2019; 237: e117808.

34.Fan S, Yan J, Sha J. Innovation and economic growth in the mining industry: evidence from China’s listed companies. Resources Policy. 2017; 54: 25–42.

35.Hou Y, Long R, Chen H, Zhang L. Research on the sustainable development of China’s coal cities based on lock-in effect. Resources Policy. 2018; 59: 479–486.

36.Krugman P. Increasing returns and economic geography. Journal of Political Economy. 1991; 99: 483–499.

37.Krugman P, Venables AJ. The seamless world: A spatial model of international specialization (No. w5220). National Bureau of Economic Research. 1995.

38.Pflüger M. Economic geography and public policy. Journal of Economic Geography. 2004; 4; 597–603.

39.Groenewold N, Li GP, Chen AP. Inter-regional spillovers in China: The importance of common shocks and the definition of the regions. China Economic Review. 2008; 19: 32–52.

40.Tian L, Wang HH, Chen Y. Spatial externalities in China regional economic growth. China Economic Review. 2010; 21: S20–S31.

41.Collet J, Ielpo F. Sector spillovers in credit markets. Journal of Banking & Finance. 2018; 94: 267–278.

42.Koutmos D. Return and volatility spillovers among cryptocurrencies. Economics Letters. 2018; 173: 122–127.

43.Guo Y, Tong L, Mei L. The effect of industrial agglomeration on green development efficiency in Northeast China since the revitalization. Journal of Cleaner Production. 2020; 258: e120584.

44.Zhang S, Wu Z, Wang Y, Hao Y. Fostering green development with green finance: An empirical study on the environmental effect of green credit policy in China. Journal of Environmental Management. 2021; 296: e113159.

45.Zheng X, Yu Y, Wang J, Deng H. Identifying the determinants and spatial nexus of provincial carbon intensity in China: A dynamic spatial panel approach. Regional Environmental Change. 2014; 14: 1651–1661.

46.Giupponi C, Gain AK. Integrated spatial assessment of the water, energy and food dimensions of the sustainable development goals. Regional Environmental Change. 2017; 17: 1881–1893.

47.Wang Y, Luo X, Chen W, Zhao M, Wang B. Exploring the spatial effect of urbanization on multi-sectoral CO2 emissions in China. Atmospheric Pollution Research. 2019; 10: 1610–1620.

48.Li B, Wu S. Effects of local and civil environmental regulation on green total factor productivity in China: a spatial Durbin econometric analysis. Journal of Cleaner Production. 2017; 153: 342–353.

49.Zhang J, Chang Y, Zhang L, Li D. Do technological innovations promote urban green development?—A spatial econometric analysis of 105 cities in China. Journal of Cleaner Production. 2018; 182: 395–403.

50.Su Y, Yu Y. Spatial association effect of regional pollution control. Journal of Cleaner Production. 2019; 213: 540–552.

51.Cheng X, Long R, Chen H, Li Q. Coupling coordination degree and spatial dynamic evolution of a regional green competitiveness system–A case study from China. Ecological Indicators. 2019; 104: 489–500.

52.Feng C, Wang M. Journey for green development transformation of China’s metal industry: A spatial econometric analysis. Journal of Cleaner Production. 2019; 225: 1105–1117.

**Comment #3:**

Table 1: The basis for index selection needs to be given. In addition, positive indicators and negative indicators need to be marked.

**Response #3:**

Thanks for this thoughtful comment. In the revised manuscript, we have offered the reasons and supportive literature for selecting the indexes, as

*“In line with the scientific, systematic, practical, and comparability principles and the dimensions identified in previous literature [2,4,40], green development level was evaluated on economic (i.e., green economic growth), environmental (i.e., environmental resource capacity), and social (i.e., governmental policy support) dimensions. The economic dimension must fully consider the unification of economic development, green productivity, industrial structure, innovation capability, and economic openness [4,19]. Therefore, our evaluation system includes 17 indicators such as GDP per capita, energy consumption per unit of GDP, CO2 emissions per unit of GDP, the proportion of transaction value in technical markets in GDP, and the labor productivity of different industries. The environmental dimension should include indicators of environmental greening, pollution prevention, and carbon balance [4,19]. Accordingly, this study applied 15 indicators such as forest coverage, proportion of nature reserve area to land area, proportion of CO2 emissions to land area, CO2 emissions per capita, consumption of chemical fertilizers, and pesticide use per unit of cultivated land area. The social dimension consists of 14 indicators, such as proportion of investment in environmental pollution control to GDP, urban green space per capita, and urban sewage treatment rates, covering aspects of industrial pollution control and infrastructure construction [4,19].”*

Please also refer to Line 128–144, Page 7 in the revised manuscript.

***References:***

2.Yang Y, Guo H, Chen L, Liu X, Gu M, Ke X. Regional analysis of the green development level differences in Chinese mineral resource-based cities. Resources Policy. 2019; 61: 261–272.

4.Li S, Liu J, Hu X. A three-dimensional evaluation model for green development: evidence from Chinese provinces along the belt and road. Environment, Development and Sustainability, 2022; in press. https://doi.org/10.1007/s10668-022-02542-w.

19.Wang M, Feng C. Revealing the pattern and evolution of global green development between different income groups: A global meta-frontier by-production technology approach. Environmental Impact Assessment Review. 2021; 89: e106600.

40.Tian L, Wang HH, Chen Y. Spatial externalities in China regional economic growth. China Economic Review. 2010; 21: S20–S31.

Additionally, positive indicators and negative indicators have been marked in Table 1 in the revised manuscript, as follows.

**Table 1. Evaluation index system of China’s green development**

| **Index** | **Calculation** | **Unit** | **Direction** |
| --- | --- | --- | --- |
| **Green economic growth** | | | |
| A1: GDP per capita | GDP/Total population | Yuan | + |
| A2: Energy consumption per unit of GDP | Total energy consumption/GDP | Ton by standard coal/ten thousand yuan | – |
| A3: Proportion of non-fossil energy consumption | Non-fossil energy consumption/Total energy consumption | % | + |
| A4: CO_2_ emissions per unit of GDP | CO_2_ emissions/GDP | Ton/ten thousand yuan | – |
| A5: SO_2_ emissions per unit of GDP | SO_2_ emissions/GDP | Ton/ten thousand yuan | – |
| A6: COD (Chemical Oxygen Demand) emissions per unit of GDP | Discharge amount of COD/GDP | Ton/ten thousand yuan | – |
| A7: NO*x* emissions per unit of GDP | NO*x* emissions/GDP | Ton/ten thousand yuan | – |
| A8: Proportion of transaction value in technical markets in GDP | Transaction value in technical markets/GDP | % | + |
| A9: Labor productivity of primary industry | Value-added of primary industry/Employment in primary industry | Yuan per capita | + |
| A10: Land output rate | Gross agricultural output value/Sown area of farm crops | % | + |
| A11: Irrigation saving rate | Water-saving irrigated area/Irrigated area | % | + |
| A12: Proportion of irrigation area | Irrigated area/Area of cultivated land | % | + |
| A13: Labor productivity of secondary industry | Value-added of secondary industry/Employment in secondary industry | Yuan per capita | + |
| A14: Water consumption per unit industrial added value | Industrial water consumption/Industrial added value | Ton/yuan | + |
| A15: Proportion of added value in the tertiary industry | Value-added of tertiary industry/GDP | % | + |
| A16: Proportion of employment in the tertiary industry | Employment of tertiary industry/Total employment | % | + |
| A17: Labor productivity of tertiary industry | Value-added of tertiary industry/Employment in the tertiary industry | % | + |
| **Environmental resource capacity** | | | |
| B1: Water resources per capita | Total water resources/Total population | m^3^/person | + |
| B2: Forest area per capita | Forest area/Total population | m^2^/person | + |
| B3: Forest coverage | Forest area/Land area | % | + |
| B4: Proportion of nature reserves area to land area | Nature reserve area/Land area | % | + |
| B5: Wetland coverage | Area of wetland/Land area | % | + |
| B6: Proportion of CO_2_ emissions to land area | CO_2_ emissions/Land area | kg/mu* | – |
| B7: CO_2_ emissions per capita | CO_2_ emissions/Total population | kg per capita | – |
| B8: SO_2_ emissions per unit land area | SO_2_ emissions/ Land area | kg/hectare | – |
| B9: SO_2_ emissions per capita | SO_2_ emissions/Total population | kg per capita | – |
| B10: COD emissions per unit land area | COD emissions/Land area | kg/mu | – |
| B11: COD emissions per capita | COD emissions/Total population | kg per capita | – |
| B12: Ammonia nitrogen emissions per unit land area | Ammonia nitrogen emissions/Land area | kg/mu | – |
| B13: Ammonia nitrogen emissions per capita | Ammonia nitrogen emissions/Total population | kg per capita | – |
| B14: Consumption of chemical fertilizers per unit of cultivated land area | Consumption of chemical fertilizers/Area of cultivated land | kg/mu | – |
| B15: Pesticide use per unit of cultivated land area | Pesticide usage/Area of cultivated land | kg/mu | – |
| **Governmental policy support** | | | |
| C1: Proportion of investment in environmental pollution control to GDP | Investment in environmental pollution treatment/GDP | % | + |
| C2: Completed amount of investment in returning grain plots to forests | Forestry investment completed/Area of cultivated land | Yuan/mu | + |
| C3:Urban green space per capita | Green area/Total urban population | % | + |
| C4:Urban sewage treatment rate | Total quantity of urban wastewater treated/Urban wastewater discharged | % | + |
| C5: Hazard-free treatment rate of urban household waste | Total quantity of urban household waste hazard-free treated/Urban household waste | % | + |
| C6: Number of public transport passengers per 10,000 urban residents | Number of public vehicles in operation (buses, trolleybuses, etc.)/Total urban population | Number per capita | + |
| C7: Greening coverage rate of built-up area | Green area of urban built-up area/Built-up area | % | + |
| C8: Newly increased afforestation area per capita | Area of afforestation/Total population | m^2^ per capita | + |
| C9: Urban green park space per capita | Green park areas/Total urban population | m^2^ per capita | + |
| C10: Proportion of R&D internal expenditure in fixed asset investment | Intramural expenditure on R&D/Investment in fixed assets | % | + |
| C11: Degree of industrial upgrade | Tertiary industry output value/Secondary industry output value | % | + |
| C12: Education expenditure per capita | Expenditure for education/Total population | Yuan | + |
| C13: Number of doctors per capita | Number of doctors/Total population | Number per capita | + |
| C14: Popularity of toilets in rural areas | Number of households using sanitary toilets/Total peasant household | % | + |

Note: *Mu* is the traditional unit for measuring land area in China. One *mu* equals 0.067 *hectares*.

**Comment #4:**

What is the meaning of Figure 1? Is it just showing the complex network structure? If so, it is recommended to delete.

**Response #4:**

Thanks for this thoughtful comment. As you mentioned, the primary purpose of using Figure 1 in the original manuscript is to show the complex network structure. We have deleted this figure in the revised manuscript, as R1 suggests.

**Comment #5:**

Figure 2 needs to be explained. Different line types should be used to indicate the different degrees of overflow and reception.

**Response #5:**

Thanks for this considerate comment. In the revised manuscript, we modified Figure 2 by using different line types to indicate the degrees of internal and spillover relations. For internal relations, we used solid lines with arrows generating and pointing to the same block. We used dotted lines with different arrow directions for spillover relations of overflow and reception. See Figure 1 in the revised manuscript as follows.

Fig 1. Four blocks and their spillover relations

We further offered explanations for this figure:

*“The four blocks of provinces and their spillover relations are summarized in Fig 1. We name each block according to its feature heterogeneity in spillover density and intensity. The definition of each block is in accordance with its feature heterogeneity in extrovert/introvert spillover intensity and green development level. If the extrovert spillover intensity is significantly greater than the introvert spillover intensity, and the green development level of the members of the block is high, the block is a net spillover block; otherwise, it is a net beneficial block. Suppose the extrovert spillover intensity of the block is close to the introvert spillover intensity. In that case, a block with a relatively high green development level is a broker block, and a block with a relatively low green development level is a two-way spillover block.”*

Please also refer to Line 369–378, Page 24 in the revised manuscript.

**Comment #6:**

It is suggested to delete the conclusion part and change the discussion part into conclusion.

**Response #6:**

Thanks for this constructive suggestion. We have reorganized the content of the discussions and conclusion, as R1 suggests. The revised content is as follows.

*“****Key findings***

*Using provincial data on the green development index system from 2006 to 2019, this study provides an overview of the spatial network characteristics of China’s green development and offers a number of interesting findings.*

*Holistically, the spatial correlation network of China’s green development at the provincial level presents a complex yet stable network structure. Almost all provinces are involved in the extrovert and introvert spillovers of green development. Provinces are all tightly related to each other with a weak hierarchy, and so the overall spatial network presents a typical small-world characteristic. Although previous research has mentioned the spatial correlations that exist in China’s green development [43,44], the current study is among the first to elucidate such network characteristics.*

*In the core–periphery relationship at the provincial level, Hunan, Tianjin, Zhejiang, Henan, and Xinjiang lie at the center of the green development network, playing critical roles in China’s overall green development. Meanwhile, Jiangxi, Heilongjiang, Jilin, Shaanxi, and Sichuan are at the network’s periphery. These findings echo previous claims of geographical heterogeneity in green development [6,7,19-21,25] at the provincial rather than the city level. However, our findings are considerably different from previous research that suggested a holistic developing advantage of eastern areas over central or western areas [4,8-10]. Specifically, we find that China’s green development network can be categorized into four heterogeneous blocks: a net spillover block, a net beneficial block, a broker block, and a two-way spillover block. Each block plays its comparative advantage in this spatially correlated network, and their linkages with each other become accumulatively prominent. The blocks incorporate provinces without prominent geographical adjacency; that is, the core–periphery role of a province in the overall spatial network of green development is considerably more complex than can be captured by a simple taxonomy based on geographical location.*

*Finally, regional differences in green economic growth, government policy support, geographic distance, and geographic adjacency are significant factors impacting the shaping of China’s green development network. Interestingly, the less difference there is in provinces’ green economic growth, the more favorable the conditions for forming a spatial correlation network. One explanation is that a similar degree of green economic development benefits the inter-regional transfer of green production factors and promotes the diffusion of energy conservation and environmental protection technologies among regions. The potential capacity of environmental resources has no significant effect, whereas government policy support positively impacts the green development network. Unlike previous research [45-47], the present study finds no significant impact of environmental resource capacity in shaping the green development network. However, consistent with studies like [28], our findings confirm the importance of government policy support in green development. Policies such as environmental regulation may motivate firms to introduce green production technologies from other regions, thereby strengthening inter-regional connections and promoting the formation of the green development network.*

***Policy recommendations***

*We put forward three recommendations for policymakers based on this study’s findings. First, policymakers should fully understand the spatial characteristics of green development, seek innovative ideas for realizing green development, and promote the effective transformation of green development policy. The improvement of green practices depends on more than the region’s economic development, environmental protection, and energy consumption structure; for instance, the levels of other regions’ green development have a role to play. The spatial correlation effect enables inter-regional collaboration in the process of green development. However, the spatial correlation of green development is not as simple as the province–province relationship; instead, there is a complex and multi-directed spillover relationship between multiple provinces. Therefore, it is necessary for both central and local governments to fully realize and leverage the spatial effects of green development. With the development of economic and technological connections among provinces in China, the spatial correlation of green development has become increasingly close. However, even with 267 relationships in the network, the relations between provinces remain weak. Hence, we should pay more attention to strengthening the spatial correlation of China’s green development. It is worth reiterating that when SNA is used, the identification of relationships is not limited to geographically adjacent regions but extends to geographically distant regions.*

*Second, the current study advocates caution and specificity in environmental regulation policy-making. The findings indicate that regional differences in green economic growth and government policy support are the main factors affecting green development’s spatial correlation. Underdeveloped areas tend to formulate relatively loose environmental regulation policies to achieve high-speed economic development. Consequently, high-polluting firms tend to transfer from areas with strict environmental regulation to areas with less environmental regulation, resulting in the aggravation of pollution in the latter areas. This “race to the bottom” presents a challenge for inter-regional cooperation on China’s green development. Nevertheless, if a unified environmental regulation policy is set without regard to each region’s heterogeneous economic and environmental characteristics, the overall level of green development will be less efficient. Thus, we conclude that the formulation of environmental regulation policies should take into account not only the unity of policies but also the differences between provinces in level of economic development and energy consumption structure. Only then can policy-making avoid the aggravation of pollution or a race to the bottom in relation to environmental regulation.*

*Third, policymakers should make full use of government control and market mechanisms in promoting green development. On the one hand, policymakers should actively take measures to improve the green development level of undeveloped regions. For instance, governmental regulation should continue to reduce the provincial gaps in economic, technological, and industrial development, thus strengthening inter-provincial green development technology connectivity and promoting the overall improvement of China’s green development level. On the other hand, policymakers should make full use of market powers to reduce administrative intervention and use market mechanisms such as competition and financial means to strengthen mutual communication between the core and peripheral areas. They should prioritize support for the latter regions to reduce China’s spatial imbalance of green development.*

***Limitations and future research directions***

*This study has a number of limitations that can inform future research. First, we investigated the spatial characteristics of China’s green development in terms of green development levels. Future research can focus on the same topic from the green development efficiency perspective (e.g., developing the green total factor productivity index). Second, this study used the objective entropy weight method to integrate the multiple indicators of green development. This method does not take into account the relative importance of indicators or the subjective intentions of decision makers; it is also limited to dealing with indicators with drastic fluctuations. Future studies should seek alternative methods. Third, this study investigated the spatial correlation, network, and spillover characteristics of China’s green development using the green development level index integrated by 55 microscopic indicators. The spatial characteristics of each microscopic feature await in-depth investigation in future studies. Fourth, this study used 13 years of data to describe the spatial correlation, network, and spillover characteristics of China’s provincial green development levels. The longer the data span, the more accurate the assessment will be, and researchers should in future extend the time span of the data.”*

Please also refer to Line 428–530, Page 28–33 in the revised manuscript.

***References:***

4.Li S, Liu J, Hu X. A three-dimensional evaluation model for green development: evidence from Chinese provinces along the belt and road. Environment, Development and Sustainability, 2022; in press. <https://doi.org/10.1007/s10668-022-02542-w.>

6.Wu H, Li Y, Hao Y, Ren S, Zhang P. Environmental decentralization, local government competition, and regional green development: evidence from China. Science of The Total Environment, 2020; 708: e135085.

7.Huang H, Mo R, Chen X. New patterns in China’s regional green development: An interval Malmquist–Luenberger productivity analysis. Structural Change and Economic Dynamics. 2021; 58: 161–173.

8.Lin B, Benjamin NI. Green development determinants in China: A non-radial quantile outlook. Journal of Cleaner Production. 2017; 162: 764–775.

9.Zhou L, Zhou C, Che L, Wang B. Spatio-temporal evolution and influencing factors of urban green development efficiency in China. Journal of Geographical Sciences. 2020; 30(5): 724–742.

10.Yang Y, Guo H, Chen L, Liu X, Gu M, Ke X. Regional analysis of the green development level differences in Chinese mineral resource-based cities. Resources Policy. 2019; 61: 261–272.

19.Wang M, Feng C. Revealing the pattern and evolution of global green development between different income groups: A global meta-frontier by-production technology approach. Environmental Impact Assessment Review. 2021; 89: e106600.

20.Wang Y, Hu H, Dai W, Burns K. Evaluation of industrial green development and industrial green competitiveness: Evidence from Chinese urban agglomerations. Ecological Indicators. 2021; 124: e107371.

21.Chen C, Ge C. Green development assessment for countries along the belt and road. Journal of environmental management. 2020; 263: e110344.

25.Wang H, He D, Yu J. Privacy-preserving incentive and rewarding scheme for crowd computing in social media. Information Sciences. 2019; 470: 15–27.

28.Jiang Z, Wang Z, Li Z. The effect of mandatory environmental regulation on innovation performance: Evidence from China. Journal of Cleaner Production. 2018; 203: 482–491.

43.Guo Y, Tong L, Mei L. The effect of industrial agglomeration on green development efficiency in Northeast China since the revitalization. Journal of Cleaner Production. 2020; 258: e120584.

44.Zhang S, Wu Z, Wang Y, Hao Y. Fostering green development with green finance: An empirical study on the environmental effect of green credit policy in China. Journal of Environmental Management. 2021; 296: e113159.

45.Zheng X, Yu Y, Wang J, Deng H. Identifying the determinants and spatial nexus of provincial carbon intensity in China: A dynamic spatial panel approach. Regional Environmental Change. 2014; 14: 1651–1661.

46.Giupponi C, Gain AK. Integrated spatial assessment of the water, energy and food dimensions of the sustainable development goals. Regional Environmental Change. 2017; 17: 1881–1893.

47.Wang Y, Luo X, Chen W, Zhao M, Wang B. Exploring the spatial effect of urbanization on multi-sectoral CO2 emissions in China. Atmospheric Pollution Research. 2019; 10: 1610–1620.

**Comment #7:**

How to define block1, 2, 3 and 4? The description of block2 and 3 is not rigorous.

**Response #7:**

Thanks for this considerate comment. In the revised manuscript, we have added a more vivid *explanation of these four blocks through Tables 4 and 5, as follows.*

*“For the BMA, the current study divides the 30 sample provinces into four blocks using the α-density index evaluation method suggested by Wasserman and Faust [73]. The blocks’ α-density matrix and image matrix are shown in Table 4. Block I contains nine provinces: Beijing, Guizhou, Yunnan, Zhejiang, Sichuan, Liaoning, Jiangxi, Hainan, and Ningxia. Block II includes seven provinces: Hunan, Inner Mongolia, Hubei, Fujian, Shanghai, Henan, and Guangxi. Block III consists of eight provinces: Anhui, Hebei, Shanxi, Guangdong, Chongqing, Shandong, Tianjin, Gansu. Block IV consists of six provinces: Heilongjiang, Jiangsu, Shaanxi, Qinghai, Jilin, Xinjiang.*

*Table 4. Relationships between blocks: spillover density matrix and image matrix*

| *Block* | *Density matrix* | | | | *Image matrix* | | | |
| --- | --- | --- | --- | --- | --- | --- | --- | --- |
|  | *Ⅰ* | *Ⅱ* | *Ⅲ* | *Ⅳ* | *Ⅰ* | *Ⅱ* | *Ⅲ* | *Ⅳ* |
| *Ⅰ* | *0.222* | *0.19* | *0.208* | *0.074* | *0* | *0* | *0* | *0* |
| *Ⅱ* | *0.444* | *0.214* | *0.262* | *0.262* | *1* | *0* | *1* | *1* |
| *Ⅲ* | *0.222* | *0.375* | *0.375* | *0.25* | *0* | *1* | *1* | *0* |
| *Ⅳ* | *0.389* | *0.429* | *0.125* | *0.133* | *1* | *1* | *0* | *0* |

*Notes: When the network density of each block is greater than the overall network density, the related value in the image matrix is 1; otherwise, it is 0.*

*Table 5 displays the spillover relations among the four blocks (intra- and inter-block). There were 227 spatial correlation relationships, 50 generated from provinces within a single block and 177 between different blocks. Thus we can see that the spatial correlation of green development in China occurs mainly between blocks. In terms of gross analysis, Block I sent out the most considerable spillovers, while Block IV spilled out the least. The number of extrovert spillovers received by Block I (65) was twice that received by Block IV (27). Because of the different number of provinces contained in the four blocks, it is impossible to evaluate scientifically the role of each block in the green development network at the aggregate level only. Therefore, we analyzed each block’s position in the network using the intensity index (the ratio of actual spillover to maximum spillover). Regarding the density analysis, Block II had the highest extrovert spillover intensity, while Block I had the lowest; for introvert spillover intensity, Block I had the highest, and Block IV had the lowest.*

*Table 5. Relationships between blocks: gross analysis and intensity analysis*

| *Block* | *No. of relationships*  *received* | | | | *Gross analysis* | | *Intensity analysis* | |
| --- | --- | --- | --- | --- | --- | --- | --- | --- |
|  | *Ⅰ* | *Ⅱ* | *Ⅲ* | *Ⅳ* | *Total extrovert spillover* | *Total introvert spillover* | *Extrovert spillover intensity* | *Introvert spillover*  *intensity* |
| *Ⅰ* | *16* | *12* | *15* | *4* | *31* | *65* | *0.157* | *0.328* |
| *Ⅱ* | *28* | *9* | *13* | *11* | *52* | *51* | *0.310* | *0.304* |
| *Ⅲ* | *16* | *21* | *21* | *12* | *49* | *34* | *0.266* | *0.185* |
| *Ⅳ* | *21* | *18* | *6* | *4* | *45* | *27* | *0.3* | *0.180* |

*The four blocks of provinces and their spillover relations are summarized in Fig 1. We name each block according to its feature heterogeneity in spillover density and intensity. The definition of each block is in accordance with its feature heterogeneity in extrovert/introvert spillover intensity and green development level. If the extrovert spillover intensity is significantly greater than the introvert spillover intensity, and the green development level of the members of the block is high, the block is a net spillover block; otherwise, it is a net beneficial block. Suppose the extrovert spillover intensity of the block is close to the introvert spillover intensity. In that case, a block with a relatively high green development level is a broker block, and a block with a relatively low green development level is a two-way spillover block.”*

And we have modified the definitions of these blocks through the relationships between their extrovert and introvert spillover intensities. The content is as follows.

*“Block I has the lowest extrovert spillover intensity (15.7%, mainly on Block III) and also the highest introvert spillover intensity (32.8%, mainly from Blocks Ⅱ and Ⅳ), indicating that this block’s green development is highly dependent on other provinces; it accepts noticeably more spillover relations from other blocks than it sends out to other blocks. Therefore, we name Block I the net spillover block.*

*Block II has the highest extrovert spillover intensity (31.0%, mainly on Blocks I, III, and IV) and the second-highest introvert spillover intensity (30.4%, from Blocks III and Ⅳ). That is, this block has the bidirectional spillover feature, in which it accepts spillover relations similar to those it sends out. This status indicates its core role in China’s green development network, and we therefore name Block II the broker block.*

*Block III is named the two-way overflow block because its extrovert spillover intensity (26.6%, mainly on Block II) is similar to its introvert spillover intensity (18.5%, mainly from Blocks I and II). This block receives more spillover relations from other blocks than it sends out.*

*Block IV is called the net beneficial block, as its introvert spillover intensity (18.0%, mainly from Block II) is much greater than its extrovert spillover intensity (30.0%, mainly on Blocks I and II). This implies that this block receives more spillover relations than it sends out.”*

Please also refer to Line 344–398, Page 22–26 in the revised manuscript.

**Comment #8:**

The paper is not clear as a whole. What is the logical relationship between the results of various methods? The result of entropy weight method is not found in this paper? In addition, the role of this result in the whole paper is not described.

**Response #8:**

Thanks for this constructive comment. According to R1’s questions, we have offered clearer explanations for combining various methods by adding a subsection called “overall methodology design” before introducing those methods. The content of the overall methodology design is as follows.

*“The present study utilized the spatial correlation network distribution characteristics of China’s green development. It first developed a system of 55 indicators to evaluate China’s provincial green development level, measuring the weight value of each indicator using the entropy weight method and calculating the green development index of each province using the linear weighted sum method. After running the nonlinear Granger causality test, it then applied SNA to construct the spatial association network of China’s green development, identifying both whole network and ego network characteristics. Next, it used BMA to analyze the role and status of each province in the spatial correlation network of green development. Provinces were categorized into several subgroups, and the spatial spillover characteristics among those subgroups were further discussed. QAP analysis (including QAP correlation analysis and QAP regression analysis) was then applied to investigate factors influencing the spatial spillovers in the green development network.”*

And we accordingly reorganized the section of results. More specifically, we highlight that, in order to figure out the spatial correlation network distribution characteristics of China’s green development, we should first develop a system to evaluate China’s provincial green development level. In doing this, we accomplished three steps. Step 1 is the establishment of the green development evaluation system through 55 indexes. Step 2 is to extract data from China Statistical Yearbooks and China’s Provincial Statistical Yearbooks from 2006 to 2019 and to normalize the data due to differences in each statistical index’s units. Step 3 applies the entropy weight method to decide each indicator’s importance (weight) in the green development evaluation system. More specifically,

*“Given that different indicators have different weights in the green development evaluation system, we applied the entropy weight method to decide the importance of each indicator [4,19,20]. The green development evaluation system could be established, and China’s provincial green development level could be calculated consequently based on the importance of each indicator.* *The entropy weight method is an objective weighting method that determines the objective weight according to the indicators’ variability. Generally, the more significant the degree of variation for an indicator’s value, the smaller the information entropy (the higher the information amount), and the more critical the role played by the indicator in the comprehensive evaluation. Compared with subjective weight assignment methods (e.g., the Delphi method), the entropy weight method is more accurate because it avoids the deviation introduced by human factors [53].”*

Please also refer to Line 111–123, Page 6, and Line 162–172, Page 11–12 in the revised manuscript.

***References:***

4.Li S, Liu J, Hu X. A three-dimensional evaluation model for green development: evidence from Chinese provinces along the belt and road. Environment, Development and Sustainability, 2022; in press. https://doi.org/10.1007/s10668-022-02542-w.

19.Wang M, Feng C. Revealing the pattern and evolution of global green development between different income groups: A global meta-frontier by-production technology approach. Environmental Impact Assessment Review. 2021; 89: e106600.

20.Wang Y, Hu H, Dai W, Burns K. Evaluation of industrial green development and industrial green competitiveness: Evidence from Chinese urban agglomerations. Ecological Indicators. 2021; 124: e107371.

53.Srdjevic B, Medeiros YDP, Faria AS. An Objective Multi-criteria Evaluation of Water Management Scenarios. Water Resources Management. 2004; 18: 35–54.

**Comment #9:**

Whether the network connectivity (c) index is used or not, the corresponding results are not found in the paper.

**Response #9:**

Thanks for this thoughtful comment. We want to apologize that although we used the network connectivity (C) index, we possibly missed the presentation of the corresponding results. We have modified this negligence in the subsection “Results of social network analysis” of the section “Empirical results” in the revised manuscript, as follows,

*“We examined the spatial network characteristics of China’s green development using UCINET software. The total number of relationships was 227. Table 2 presents the whole network characteristics of China’s green development. ... The network connectivity (C = 1) implies that all provinces were in the network. ...”*

Please also refer to Line 315, Page 20 in the revised manuscript.

**Response to Reviewer 2**

**Comment #1:**

This manuscript studies the spatial spillover effect and impacting factors on green development at the provincial level in China from a social network perspective, this topic is interesting, the analysis is solid. Yet a few concerns need to be addressed before publication:

**Response #1:**

Thanks for all the encouraging comments! We have carefully revised the manuscript addressing to R2’s concerns.

**Comment #2:**

The English expression of the whole manuscript is hard to read, it needs to be revised and refined. It is recommended to invite polishing institutions to provide services.

**Response #2:**

Thanks for this considerate suggestion. We have thoroughly improved the writing and have a professional copy editor proofread the manuscript.

Please also refer to the proofreading certificate attached to this response letter.

**Comment #3:**

Figure 2 is very vague, it is suggested to improve the image quality.

**Response #3:**

Thanks for this considerate comment. We have replaced Figure 2 with a clearer version, and additionally, according to R1’s suggestions, some details in this figure have been further modified.

Fig 1^[1]^. Four blocks and their spillover relations

Please also refer to Line 379–380, Page 25 in the revised manuscript.

**Comment #4:**

It is suggested to give the limitations of this study and the future research direction, especially the methods, data, influencing factors, etc.

**Response #4:**

Thanks for this constructive suggestion. We have added the following content in the section of discussions and conclusion. Limitations about the methods, data, influencing factors thus are offered, and future research directions, accordingly.

*“This study has a number of limitations that can inform future research. First, we investigated the spatial characteristics of China’s green development in terms of green development levels. Future research can focus on the same topic from the green development efficiency perspective (e.g., developing the green total factor productivity index). Second, this study used the objective entropy weight method to integrate the multiple indicators of green development. This method does not take into account the relative importance of indicators or the subjective intentions of decision makers; it is also limited to dealing with indicators with drastic fluctuations. Future studies should seek alternative methods. Third, this study investigated the spatial correlation, network, and spillover characteristics of China’s green development using the green development level index integrated by 55 microscopic indicators. The spatial characteristics of each microscopic feature await in-depth investigation in future studies. Fourth, this study used 13 years of data to describe the spatial correlation, network, and spillover characteristics of China’s provincial green development levels. The longer the data span, the more accurate the assessment will be, and researchers should in future extend the time span of the data.”*

Please also refer to Line 515–530, Page 32–33 in the revised manuscript.

**Comment #5:**

The literature review is suggested to supplement and expand.

**Response #5:**

Thanks for this suggestion. In the revised manuscript, we have added a few recent publications on green development and space spillovers. Specifically, we organize the relative literature into three aspects: 1) research on development heterogeneity of green development; 2) impact factors of green development; 3) spatial interdependence and spillover of green development. See the content below:

*“Recent research on regional green development can be divided into several aspects around development heterogeneity. First, researchers have focused on establishing an evaluation system of green development levels and efficiencies. Some researchers have developed operational measurements of regional green development level based on the analytic hierarchy process (AHP) entropy method [2-3], the three-dimensional AHP method [4], the entropy technique for order preference by similarity to ideal solution model in combination with the Theil index method [19,20], the multi-hierarchy linear summation method [21], or using a software tool to assess energies in multi-scale life cycles [22]. Other researchers have focused on establishing the green total factor productivity to depict the green development efficiency among different regions. For instance, researchers have applied data envelopment analysis, including the non-radial direction distance function method [5], the slacks-based measure (SBM) model with undesirable outputs [7], and the SBM Malmquist–Luenberger model to evaluate the green total factor productivity [7,23,24]. By evaluating those green development indexes, researchers have found an imbalance in green development among different regions. Specifically, eastern China has outpaced central and western China in green development [4,8-10], and certain cities have higher green development levels or efficiencies than other cities within the same region [6,7,19-21, 25].*

*Researchers have identified a range of impact factors of green development, with research on the effect of green development policy, especially environmental regulations, being particularly notable. The Chinese government has put forward environmental regulations in relation to both command-control and market-related policies, prioritizing sustainable development strategies. Different environmental regulations may affect green development unequally [11,13,26]. Variations in policy effectiveness may be one reason the literature offers diverse viewpoints regarding the effects of environmental regulations. For instance, Pan et al. [27] stated that environmental regulations contribute to technological advancement and energy conservation. Jiang et al. [28] found that environmental regulations function negatively for green development, inhibiting enterprises’ R&D investment, and thus impeding their green innovation practice. Comparing US versus Swiss hydropower generation regulations, Tonka [29] proposed that defects in the regulatory framework might be the reason for environmental over-exploitation. Mycoo et al. [30] concluded that regulations are ineffective when proper regulatory tools are unavailable: a governance framework should be combined with the available effective market instruments, new technologies, behavioral change, and land tenure reform. Other factors have also been highlighted, including climatic changes and human activities [14], financial activities such as green credit and renewable energy investment [15,24,31-33], public participation and regulatory compliance [16], technical progress, and innovation [7,17,18,34,35].*

*Since the “economic geography” theory was put forward [36], the spatial correlations of economic activities of agglomeration have drawn attention in many contexts [37,38], including economic development [39,40] and financial sectors [41,42]. Spatial correlations among regions also exist in the context of green development. For instance, green environment efficiency differs across regions and shows solid spatial dependence [43,44]. This spatial interdependence is prominent in specific indicators such as carbon dioxide emissions, carbon intensity, and water resources [45-47] and in single factors like environmental regulation, technological innovation, and pollution control programs [48-50], as well as the more comprehensive green development indicators of green competitiveness [51] and green total factor productivity [52].”*

Please also refer to Line 63–109, Page 4–6 in the revised manuscript.

***References:***

2.Yang Y, Guo H, Chen L, Liu X, Gu M, Ke X. Regional analysis of the green development level differences in Chinese mineral resource-based cities. Resources Policy. 2019; 61: 261–272.

3.Guo ST, Zhang WH, Liu ZJ. Research on the temporal and spatial differentiation of the provincial green development index along the “the belt and road”. Journal of Chongqing University of Arts and Sciences. 2022; in press. https://kns.cnki.net/kcms/detail/50.1182.C.20220117.1342.002.html.

4.Li S, Liu J, Hu X. A three-dimensional evaluation model for green development: evidence from Chinese provinces along the belt and road. Environment, Development and Sustainability, 2022; in press. https://doi.org/10.1007/s10668-022-02542-w.

5.Wang W, Xie HL, Lu FC, Zhang XM. Measuring the performance of industrial green development using a non-radial directional distance function approach: A Case Study of Jiangxi Province in China. Sustainability. 2017; 9(10): e1757.

6.Wu H, Li Y, Hao Y, Ren S, Zhang P. Environmental decentralization, local government competition, and regional green development: evidence from China. Science of The Total Environment, 2020; 708: e135085.

7.Huang H, Mo R, Chen X. New patterns in China’s regional green development: An interval Malmquist–Luenberger productivity analysis. Structural Change and Economic Dynamics. 2021; 58: 161–173.

8.Lin B, Benjamin NI. Green development determinants in China: A non-radial quantile outlook. Journal of Cleaner Production. 2017; 162: 764–775.

9.Zhou L, Zhou C, Che L, Wang B. Spatio-temporal evolution and influencing factors of urban green development efficiency in China. Journal of Geographical Sciences. 2020; 30(5): 724–742.

10.Yang Y, Guo H, Chen L, Liu X, Gu M, Ke X. Regional analysis of the green development level differences in Chinese mineral resource-based cities. Resources Policy. 2019; 61: 261–272.

11.Li R, Ramanathan R. Exploring the relationships between different types of environmental regulations and environmental performance: Evidence from China. Journal of Cleaner Production. 2018; 196: 1329–1340.

12.Huang J, Zhang, W; Ruan, W. Spatial spillover and impact factors of the internet finance development in China. Physica A: Statistical Mechanics and its Applications. 2019; 527: e121390.

13.Yang J, Guo H, Liu B, Shi R, Zhang B, Ye W. Environmental regulation and the Pollution Haven Hypothesis: Do environmental regulation measures matter?. Journal of Cleaner Production. 2018; 202: 993–1000.

14.Lu X. Vulnerability of water discharge of large Chinese rivers to environmental changes: an overview. Regional Environmental Change. 2004; 4: 182–191.

15.He L, Zhang L, Zhong Z, Wang D, Wang F. Green credit, renewable energy investment and green economy development: Empirical analysis based on 150 listed companies of China. Journal of Cleaner Production. 2019; 208: 363–372.

16.Fu J, Geng Y. Public participation, regulatory compliance and green development in China based on provincial panel data. Journal of Cleaner Production. 2019; 230: 1344–1353.

17.Tao F, Zhang H, Hu J, Xia X. Dynamics of green productivity growth for major Chinese urban agglomerations. Applied Energy. 2017; 196: 170–179.

18.Li W, Wang J, Chen R, Xi Y, Liu S, Wu F, Masoud M, Wu X. Innovation-driven industrial green development: The moderating role of regional factors. Journal of Cleaner Production. 2019; 222: 344–354.

19.Wang M, Feng C. Revealing the pattern and evolution of global green development between different income groups: A global meta-frontier by-production technology approach. Environmental Impact Assessment Review. 2021; 89: e106600.

20.Wang Y, Hu H, Dai W, Burns K. Evaluation of industrial green development and industrial green competitiveness: Evidence from Chinese urban agglomerations. Ecological Indicators. 2021; 124: e107371.

21.Chen C, Ge C. Green development assessment for countries along the belt and road. Journal of environmental management. 2020; 263: e110344.

22.Allende AL, Stephan A. Life cycle embodied, operational and mobility-related energy and greenhouse gas emissions analysis of a green development in Melbourne, Australia. Applied Energy. 2022; 305: e117886.

23.Streimikis J, Miao Z, Balezentis T. Creation of climate-smart and energy-efficient agriculture in the European Union: pathways based on the frontier analysis. Business Strategy and the Environment. 2021; 30(1): 576–589.

24.Yu C, Wenxin L, Khan SU, Yu C, Jun Z, Yue D, Zhao M. Regional differential decomposition and convergence of rural green development efficiency: evidence from China. Environmental Science and Pollution Research. 2020; 27(18): 22364–22379.

25.Wang H, He D, Yu J. Privacy-preserving incentive and rewarding scheme for crowd computing in social media. Information Sciences. 2019; 470: 15–27.

26.Shen N, Liao H, Deng R, Wang Q. Different types of environmental regulations and the heterogeneous influence on the environmental total factor productivity: Empirical analysis of China’s industry. Journal of Cleaner Production. 2019; 211: 171–184.

27.Pan X, Ai B, Li C, Pan X, Yan Y. Dynamic relationship among environmental regulation, technological innovation and energy efficiency based on large scale provincial panel data in China. Technological Forecasting and Social Change. 2017; 144: 428–435.

28.Jiang Z, Wang Z, Li Z. The effect of mandatory environmental regulation on innovation performance: Evidence from China. Journal of Cleaner Production. 2018; 203: 482–491.

29.Tonka L. Hydropower license renewal and environmental protection policies: a comparison between Switzerland and the USA. Regional Environmental Change. 2015; 15: 539–548.

30.Mycoo MA, Griffith-Charles C, Lalloo S. Land management and environmental change in small-island-developing states: the case of St. Lucia. Regional Environmental Change. 2017; 17: 1065–1076.

31.Feng C, Wang M. Journey for green development transformation of China’s metal industry: A spatial econometric analysis. Journal of Cleaner Production, 2019; 225: 1105–1117.

32.Dong XJ, Shi T. The green development efficiency and influencing factors of central plains urban agglomeration. Regional Economic Review. 2018; 5: 116–122.

33.Yuan H, Zhang T, Feng Y, Liu Y, Ye X. Does financial agglomeration promote the green development in China? A spatial spillover perspective. Journal of Cleaner Production. 2019; 237: e117808.

34.Fan S, Yan J, Sha J. Innovation and economic growth in the mining industry: evidence from China’s listed companies. Resources Policy. 2017; 54: 25–42.

35.Hou Y, Long R, Chen H, Zhang L. Research on the sustainable development of China’s coal cities based on lock-in effect. Resources Policy. 2018; 59: 479–486.

36.Krugman P. Increasing returns and economic geography. Journal of Political Economy. 1991; 99: 483–499.

37.Krugman P, Venables AJ. The seamless world: A spatial model of international specialization (No. w5220). National Bureau of Economic Research. 1995.

38.Pflüger M. Economic geography and public policy. Journal of Economic Geography. 2004; 4; 597–603.

39.Groenewold N, Li GP, Chen AP. Inter-regional spillovers in China: The importance of common shocks and the definition of the regions. China Economic Review. 2008; 19: 32–52.

40.Tian L, Wang HH, Chen Y. Spatial externalities in China regional economic growth. China Economic Review. 2010; 21: S20–S31.

41.Collet J, Ielpo F. Sector spillovers in credit markets. Journal of Banking & Finance. 2018; 94: 267–278.

42.Koutmos D. Return and volatility spillovers among cryptocurrencies. Economics Letters. 2018; 173: 122–127.

43.Guo Y, Tong L, Mei L. The effect of industrial agglomeration on green development efficiency in Northeast China since the revitalization. Journal of Cleaner Production. 2020; 258: e120584.

44.Zhang S, Wu Z, Wang Y, Hao Y. Fostering green development with green finance: An empirical study on the environmental effect of green credit policy in China. Journal of Environmental Management. 2021; 296: e113159.

45.Zheng X, Yu Y, Wang J, Deng H. Identifying the determinants and spatial nexus of provincial carbon intensity in China: A dynamic spatial panel approach. Regional Environmental Change. 2014; 14: 1651–1661.

46.Giupponi C, Gain AK. Integrated spatial assessment of the water, energy and food dimensions of the sustainable development goals. Regional Environmental Change. 2017; 17: 1881–1893.

47.Wang Y, Luo X, Chen W, Zhao M, Wang B. Exploring the spatial effect of urbanization on multi-sectoral CO2 emissions in China. Atmospheric Pollution Research. 2019; 10: 1610–1620.

48.Li B, Wu S. Effects of local and civil environmental regulation on green total factor productivity in China: a spatial Durbin econometric analysis. Journal of Cleaner Production. 2017; 153: 342–353.

49.Zhang J, Chang Y, Zhang L, Li D. Do technological innovations promote urban green development?—A spatial econometric analysis of 105 cities in China. Journal of Cleaner Production. 2018; 182: 395–403.

50.Su Y, Yu Y. Spatial association effect of regional pollution control. Journal of Cleaner Production. 2019; 213: 540–552.

51.Cheng X, Long R, Chen H, Li Q. Coupling coordination degree and spatial dynamic evolution of a regional green competitiveness system–A case study from China. Ecological Indicators. 2019; 104: 489–500.

52.Feng C, Wang M. Journey for green development transformation of China’s metal industry: A spatial econometric analysis. Journal of Cleaner Production. 2019; 225: 1105–1117.

**Comment #6:**

In general, there is not enough explanation or analysis in Empirical Results as they are very important sections for readers’ understanding.

**Response #6:**

Thanks for this constructive comment. In the revised manuscript, we put much effort into developing more detailed explanations of the empirical results, as follows.

*“****Results of social network analysis***

*We examined the spatial network characteristics of China’s green development using UCINET software. The total number of relationships was 227. Table 2 presents the whole network characteristics of China’s green development. The network density was relatively high (D = 0.2609), indicating that a complex network structure represents green development in China. The network connectivity (C = 1) implies that all provinces were in the network. The high network efficiency (E = 0.574) indicates redundant relationships in the network or that the network structure was relatively stable, and the nodes were tightly connected with each other. The low network hierarchy degree (H = 0.067) shows that the hierarchy in the network had gradually been weakened, and that the provinces with different green development levels were possibly having spillover effects on other provinces. The clustering coefficients and average path length (A = 0.892, AL = 1.834) imply that China’s green development network reflects the prominent small-world characteristics. Altogether, the results show that China’s green development has a complex network structure.*

*[Table 2 here]*

*Table 3 shows the ego network characteristics of each province. The point centrality (*${PC}_{i}$*) has a mean value of 46.437, much larger than its standard deviation (9.868). Similarly, the closeness centrality (*$C_{{AP}_{i}}^{-1}$*) has a mean value of 65.382, much larger than its standard deviation (4.243), and the betweenness centrality (*${BC}_{i}$*) has a mean value of 1.913 and a standard deviation of 1.180. These results indicate that there is little variance among the green development network provinces. Moreover, the centralizations of point centrality, closeness centrality, and betweenness centrality are 17.981, 27.157, and 13.145, respectively, which taken together suggest that the green development network represents a spatially divergent trend. Specifically, the provinces of Hunan, Tianjin, Zhejiang, Henan, and Xinjiang ranked highest in point centrality, whereas Jiangxi, Heilongjiang, Jilin, Shaanxi, and Sichuan ranked the lowest. For closeness centrality, Hunan, Tianjin, Zhejiang, Henan, and Xinjiang ranked highest, and Jiangxi, Heilongjiang, Jilin, Shaanxi, and Sichuan ranked lowest. Similarly, for betweenness centrality, Hunan, Tianjin, Henan, Guangxi, and Yunnan ranked highest, and Jiangxi, Jilin, Shaanxi, Shanghai, and Shandong ranked lowest. The general picture is one of Hunan, Tianjin, Zhejiang, Henan, and Xinjiang occupying core positions in China’s green development network, with Jiangxi, Jilin, Shaanxi, Heilongjiang, and Sichuan occupying peripheral positions.*

*[Table 3 here]*

***Results of block model analysis***

*For the BMA, the current study divides the 30 sample provinces into four blocks using the α-density index evaluation method suggested by Wasserman and Faust [73]. The blocks’ α-density matrix and image matrix are shown in Table 4. Block I contains nine provinces: Beijing, Guizhou, Yunnan, Zhejiang, Sichuan, Liaoning, Jiangxi, Hainan, and Ningxia. Block II includes seven provinces: Hunan, Inner Mongolia, Hubei, Fujian, Shanghai, Henan, and Guangxi. Block III consists of eight provinces: Anhui, Hebei, Shanxi, Guangdong, Chongqing, Shandong, Tianjin, Gansu. Block IV consists of six provinces: Heilongjiang, Jiangsu, Shaanxi, Qinghai, Jilin, Xinjiang.*

*[Table 4 here]*

*Table 5 displays the spillover relations among the four blocks (intra- and inter-block). There were 227 spatial correlation relationships, 50 generated from provinces within a single block and 177 between different blocks. Thus we can see that the spatial correlation of green development in China occurs mainly between blocks. In terms of gross analysis, Block I sent out the most considerable spillovers, while Block IV spilled out the least. The number of extrovert spillovers received by Block I (65) was twice that received by Block IV (27). Because of the different number of provinces contained in the four blocks, it is impossible to evaluate scientifically the role of each block in the green development network at the aggregate level only. Therefore, we analyzed each block’s position in the network using the intensity index (the ratio of actual spillover to maximum spillover). Regarding the density analysis, Block II had the highest extrovert spillover intensity, while Block I had the lowest; for introvert spillover intensity, Block I had the highest, and Block IV had the lowest.*

*[Table 5 here]*

*The four blocks of provinces and their spillover relations are summarized in Fig 1. We name each block according to its feature heterogeneity in spillover density and intensity. The definition of each block is in accordance with its feature heterogeneity in extrovert/introvert spillover intensity and green development level. If the extrovert spillover intensity is significantly greater than the introvert spillover intensity, and the green development level of the members of the block is high, the block is a net spillover block; otherwise, it is a net beneficial block. Suppose the extrovert spillover intensity of the block is close to the introvert spillover intensity. In that case, a block with a relatively high green development level is a broker block, and a block with a relatively low green development level is a two-way spillover block.*

*[Figure 1 here]*

*Block I has the lowest extrovert spillover intensity (15.7%, mainly on Block III) and also the highest introvert spillover intensity (32.8%, mainly from Blocks Ⅱ and Ⅳ), indicating that this block’s green development is highly dependent on other provinces; it accepts noticeably more spillover relations from other blocks than it sends out to other blocks. Therefore, we name Block I the net spillover block.*

*Block II has the highest extrovert spillover intensity (31.0%, mainly on Blocks I, III, and IV) and the second-highest introvert spillover intensity (30.4%, from Blocks III and Ⅳ). That is, this block has the bidirectional spillover feature, in which it accepts spillover relations similar to those it sends out. This status indicates its core role in China’s green development network, and we therefore name Block II the broker block.*

*Block III is named the two-way overflow block because its extrovert spillover intensity (26.6%, mainly on Block II) is similar to its introvert spillover intensity (18.5%, mainly from Blocks I and II). This block receives more spillover relations from other blocks than it sends out.*

*Block IV is called the net beneficial block, as its introvert spillover intensity (18.0%, mainly from Block II) is much greater than its extrovert spillover intensity (30.0%, mainly on Blocks I and II). This implies that this block receives more spillover relations than it sends out.*

***Result of quadratic assignment procedure analysis***

*QAP correlation analysis shows the relationship between a single influencing factor and the green development’s overall spatial relationship. As listed in Table 6, despite the regional differences in environmental resource capacity, all other factors were significantly correlated (at the 1% level) to the overall spatial relationship of green development. Specifically, the regional difference in the greening rate of economic growth was negatively correlated to the overall spatial relationship of green development. In contrast, regional differences in government policy and spatial adjacency were positive. In terms of geographic distance, the coefficients of distances less than 500 km were significantly positively correlated to the overall spatial relationship of green development. Generally, government policy support, spatial adjacency, and geographic distance positively affected the overall spatial relationship of green development.*

*[Table 6 here]*

*QAP regression analysis shows the impact of different factors in one province on the green development of the other provinces. As listed in Table 7, for the regression model on geographic distance, despite regional differences in environmental resource capacity, all other factors imposed significant effects (at the 5% level) on the green development network. Specifically, the impact of regional differences in green economic growth on regional differences in green development was negative (β = –0.023, p = 0.037). For regional differences in government policy support (β = 0.214, p = 0.020) and distances shorter than 500 km (β = 0.255, p = 0.023 for 0–250 km; β = 0.132, p = 0.011 for 250–500 km), all effects were positive. Additionally, coefficients of geographic distance fell as the distance rose, implying that the influence of geographic distance on regional differences in green development decreased when the distance increased. For the regression model on spatial adjacency, Table 8 confirms robustness, as there were no obvious changes in the coefficients of the independent variables.*

*[Table 7 and Table 8 here]”*

Please also refer to Line 310–426, Page 20–28 in the revised manuscript.

***Reference:***

73.Wasserman S, Faust K. Social network analysis: Methods and applications. Cambridge University Press; 1994.

**Comment #7:**

An explanation of why the authors select these 55 fundamental indicators should be provided.

**Response #7:**

Thanks for this thoughtful comment. In the revised manuscript, we have offered the reasons and supportive literature for selecting the indexes, as

*“In line with the scientific, systematic, practical, and comparability principles and the dimensions identified in previous literature [2,4,40], green development level was evaluated on economic (i.e., green economic growth), environmental (i.e., environmental resource capacity), and social (i.e., governmental policy support) dimensions. The economic dimension must fully consider the unification of economic development, green productivity, industrial structure, innovation capability, and economic openness [4,19]. Therefore, our evaluation system includes 17 indicators such as GDP per capita, energy consumption per unit of GDP, CO2 emissions per unit of GDP, the proportion of transaction value in technical markets in GDP, and the labor productivity of different industries. The environmental dimension should include indicators of environmental greening, pollution prevention, and carbon balance [4,19]. Accordingly, this study applied 15 indicators such as forest coverage, proportion of nature reserve area to land area, proportion of CO2 emissions to land area, CO2 emissions per capita, consumption of chemical fertilizers, and pesticide use per unit of cultivated land area. The social dimension consists of 14 indicators, such as proportion of investment in environmental pollution control to GDP, urban green space per capita, and urban sewage treatment rates, covering aspects of industrial pollution control and infrastructure construction [4,19].”*

Please also refer to Line 128–144, Page 7 in the revised manuscript.

***References:***

2.Yang Y, Guo H, Chen L, Liu X, Gu M, Ke X. Regional analysis of the green development level differences in Chinese mineral resource-based cities. Resources Policy. 2019; 61: 261–272.

4.Li S, Liu J, Hu X. A three-dimensional evaluation model for green development: evidence from Chinese provinces along the belt and road. Environment, Development and Sustainability, 2022; in press. https://doi.org/10.1007/s10668-022-02542-w.

19.Wang M, Feng C. Revealing the pattern and evolution of global green development between different income groups: A global meta-frontier by-production technology approach. Environmental Impact Assessment Review. 2021; 89: e106600.

40.Tian L, Wang HH, Chen Y. Spatial externalities in China regional economic growth. China Economic Review. 2010; 21: S20–S31.

**Comment #8:**

Section Results of Block Model Analysis is suggested to expand.

**Response #8:**

Thanks for this thoughtful comment. In the revised manuscript, we have added a more vivid explanation of these four blocks through Tables 4 and 5.

*“For the BMA, the current study divides the 30 sample provinces into four blocks using the α-density index evaluation method suggested by Wasserman and Faust [73]. The blocks’ α-density matrix and image matrix are shown in Table 4. Block I contains nine provinces: Beijing, Guizhou, Yunnan, Zhejiang, Sichuan, Liaoning, Jiangxi, Hainan, and Ningxia. Block II includes seven provinces: Hunan, Inner Mongolia, Hubei, Fujian, Shanghai, Henan, and Guangxi. Block III consists of eight provinces: Anhui, Hebei, Shanxi, Guangdong, Chongqing, Shandong, Tianjin, Gansu. Block IV consists of six provinces: Heilongjiang, Jiangsu, Shaanxi, Qinghai, Jilin, Xinjiang.*

*Table 4. Relationships between blocks: spillover density matrix and image matrix*

| *Block* | *Density matrix* | | | | *Image matrix* | | | |
| --- | --- | --- | --- | --- | --- | --- | --- | --- |
|  | *Ⅰ* | *Ⅱ* | *Ⅲ* | *Ⅳ* | *Ⅰ* | *Ⅱ* | *Ⅲ* | *Ⅳ* |
| *Ⅰ* | *0.222* | *0.19* | *0.208* | *0.074* | *0* | *0* | *0* | *0* |
| *Ⅱ* | *0.444* | *0.214* | *0.262* | *0.262* | *1* | *0* | *1* | *1* |
| *Ⅲ* | *0.222* | *0.375* | *0.375* | *0.25* | *0* | *1* | *1* | *0* |
| *Ⅳ* | *0.389* | *0.429* | *0.125* | *0.133* | *1* | *1* | *0* | *0* |

*Notes: When the network density of each block is greater than the overall network density, the related value in the image matrix is 1; otherwise, it is 0.*

*Table 5 displays the spillover relations among the four blocks (intra- and inter-block). There were 227 spatial correlation relationships, 50 generated from provinces within a single block and 177 between different blocks. Thus we can see that the spatial correlation of green development in China occurs mainly between blocks. In terms of gross analysis, Block I sent out the most considerable spillovers, while Block IV spilled out the least. The number of extrovert spillovers received by Block I (65) was twice that received by Block IV (27). Because of the different number of provinces contained in the four blocks, it is impossible to evaluate scientifically the role of each block in the green development network at the aggregate level only. Therefore, we analyzed each block’s position in the network using the intensity index (the ratio of actual spillover to maximum spillover). Regarding the density analysis, Block II had the highest extrovert spillover intensity, while Block I had the lowest; for introvert spillover intensity, Block I had the highest, and Block IV had the lowest.*

*Table 5. Relationships between blocks: gross analysis and intensity analysis*

| *Block* | *No. of relationships*  *received* | | | | *Gross analysis* | | *Intensity analysis* | |
| --- | --- | --- | --- | --- | --- | --- | --- | --- |
|  | *Ⅰ* | *Ⅱ* | *Ⅲ* | *Ⅳ* | *Total extrovert spillover* | *Total introvert spillover* | *Extrovert spillover intensity* | *Introvert spillover*  *intensity* |
| *Ⅰ* | *16* | *12* | *15* | *4* | *31* | *65* | *0.157* | *0.328* |
| *Ⅱ* | *28* | *9* | *13* | *11* | *52* | *51* | *0.310* | *0.304* |
| *Ⅲ* | *16* | *21* | *21* | *12* | *49* | *34* | *0.266* | *0.185* |
| *Ⅳ* | *21* | *18* | *6* | *4* | *45* | *27* | *0.3* | *0.180* |

*The four blocks of provinces and their spillover relations are summarized in Fig 1. We name each block according to its feature heterogeneity in spillover density and intensity. The definition of each block is in accordance with its feature heterogeneity in extrovert/introvert spillover intensity and green development level. If the extrovert spillover intensity is significantly greater than the introvert spillover intensity, and the green development level of the members of the block is high, the block is a net spillover block; otherwise, it is a net beneficial block. Suppose the extrovert spillover intensity of the block is close to the introvert spillover intensity. In that case, a block with a relatively high green development level is a broker block, and a block with a relatively low green development level is a two-way spillover block.”*

And we have modified the definitions of these blocks through the relationships between their extrovert and introvert spillover intensities. The content is as follows.

*“Block I has the lowest extrovert spillover intensity (15.7%, mainly on Block III) and also the highest introvert spillover intensity (32.8%, mainly from Blocks Ⅱ and Ⅳ), indicating that this block’s green development is highly dependent on other provinces; it accepts noticeably more spillover relations from other blocks than it sends out to other blocks. Therefore, we name Block I the net spillover block.*

*Block II has the highest extrovert spillover intensity (31.0%, mainly on Blocks I, III, and IV) and the second-highest introvert spillover intensity (30.4%, from Blocks III and Ⅳ). That is, this block has the bidirectional spillover feature, in which it accepts spillover relations similar to those it sends out. This status indicates its core role in China’s green development network, and we therefore name Block II the broker block.*

*Block III is named the two-way overflow block because its extrovert spillover intensity (26.6%, mainly on Block II) is similar to its introvert spillover intensity (18.5%, mainly from Blocks I and II). This block receives more spillover relations from other blocks than it sends out.*

*Block IV is called the net beneficial block, as its introvert spillover intensity (18.0%, mainly from Block II) is much greater than its extrovert spillover intensity (30.0%, mainly on Blocks I and II). This implies that this block receives more spillover relations than it sends out.”*

Please also refer to Line 344–398, Page 22–26 in the revised manuscript.

**Comment #9:**

Section Result of Quadratic Assignment Procedure Analysis is suggested to expand and give more detail, for example, the permutations, the explanation of QAP correlation and QAP regression, etc.

**Response #9:**

Thanks for this insightful suggestion. We first expanded the methodology section by offering the permutations of QAP as follows:

*“QAP is an SNA method for studying the relationship between different relations existing in a network; that is, it examines the correlation and regression between two relationship matrices. This method is based on permutations of matrices. It compares the corresponding lattice values of pairwise matrices, provides the correlation coefficients between them, and performs a non-parametric test on the coefficients [71]. Specifically, the method consisted of three steps. First, the correlation coefficient between two known matrices was calculated: the matrix was converted into a long vector, and the correlation coefficient between them was calculated [72]. Second, the rows and columns of one matrix were randomly replaced. Then the correlation coefficient between the replaced matrix and the other matrix was calculated, and this operation was repeated many times to achieve a distribution [72]. Third, the correlation coefficient calculated in the first step was compared with the distribution achieved in the second step to check for statistically significant differences [72]. This approach allowed the influencing factors’ effects on regional spatial correlation to be investigated [12]. QAP analysis includes QAP correlation analysis and QAP regression analysis [72]. The former investigates the correlation between two relationship matrices and between an attribute and a relationship [72]. The latter examines the regression relationship between multiple matrices and the focal matrix [72].”*

Then we expanded the section on the result of quadratic assignment procedure analysis by offering the explanation of QAP correlation and QAP regression. The specific content is as follows.

*“QAP correlation analysis shows the relationship between a single influencing factor and the green development’s overall spatial relationship. As listed in Table 6, despite the regional differences in environmental resource capacity, all other factors were significantly correlated (at the 1% level) to the overall spatial relationship of green development. Specifically, the regional difference in the greening rate of economic growth was negatively correlated to the overall spatial relationship of green development. In contrast, regional differences in government policy and spatial adjacency were positive. In terms of geographic distance, the coefficients of distances less than 500 km were significantly positively correlated to the overall spatial relationship of green development. Generally, government policy support, spatial adjacency, and geographic distance positively affected the overall spatial relationship of green development.*

*[Table 6 here]*

*QAP regression analysis shows the impact of different factors in one province on the green development of the other provinces. As listed in Table 7, for the regression model on geographic distance, despite regional differences in environmental resource capacity, all other factors imposed significant effects (at the 5% level) on the green development network. Specifically, the impact of regional differences in green economic growth on regional differences in green development was negative (β = –0.023, p = 0.037). For regional differences in government policy support (β = 0.214, p = 0.020) and distances shorter than 500 km (β = 0.255, p = 0.023 for 0–250 km; β = 0.132, p = 0.011 for 250–500 km), all effects were positive. Additionally, coefficients of geographic distance fell as the distance rose, implying that the influence of geographic distance on regional differences in green development decreased when the distance increased. For the regression model on spatial adjacency, Table 8 confirms robustness, as there were no obvious changes in the coefficients of the independent variables.*

*[Table 7 and Table 8 here]”*

Please also refer to Line 283–299, Page 18–19, and Line 399–426, Page 26–28 in the revised manuscript.

***References:***

12.Huang J, Zhang, W; Ruan, W. Spatial spillover and impact factors of the internet finance development in China. Physica A: Statistical Mechanics and its Applications. 2019; 527: e121390.

71.Tobler WR. A computer movie simulating urban growth in the Detroit region. Economic Geography. 1970; 46: 234–240.

72.Cheverud JM, Dow MM, Leutenegger W. The quantitative assessment of phylogenetic constraints in comparative analyses: sexual dimorphism in body weight among primates. Evolution. 1985; 39(6): 1335–1351.

1. Figure 2 has been modified as Figure 1 as we deleted the original Figure 1 according to R1’s suggestion.
